# Rural adolescent attitudes and use of bicycle helmets in Iowa

**DOI:** 10.1186/s40621-025-00596-8

**Published:** 2025-07-14

**Authors:** Brooke L. Askelsen, Brianna J. Iverson, Devin E. Spolsdoff, Pam J. Hoogerwerf, Brenda Vergara, Charles A. Jennissen

**Affiliations:** 1 University of Iowa College of Liberal Arts and Sciences, Iowa City, IA USA; 2 Roy J. and Lucille A. Carver College of Medicine, University of Iowa, Iowa City, IA USA; 3 Department of Emergency Medicine, Roy J. and Lucille A. Carver College of Medicine, University of Iowa, Iowa City, IA USA; 4 Injury Prevention and Community Outreach, Stead Family Children’s Hospital, University of Iowa, Iowa City, IA USA; 5 Stead Family Department of Pediatrics, Roy J. and Lucille A. Carver College of Medicine, University of Iowa, Iowa City, IA USA; 6 University of Iowa Hospitals and Clinics, 200 Hawkins Dr, Iowa City, IA 52242 USA

**Keywords:** Adolescent, Bicycle, Bike, Farm, Helmet, Rural, Safety, Youth

## Abstract

**Background:**

Helmet use significantly decreases head injuries, the most common cause of bicycle-related fatalities in youth. Our objective was to determine bicycle helmet use by rural adolescents, their attitudes regarding helmets, and associated demographic factors.

**Methods:**

A convenience sample of 2022 Iowa FFA (formerly Future Farmers of America) Leadership Conference attendees completed an anonymous electronic or paper survey. After compilation in Qualtrics, descriptive, bivariate and multivariable logistic regression analyses were performed using statistical program, R.

**Results:**

1,331 adolescents 13–18 years participated. Almost three-fifths (58%) were female; 56% were 16–18 years. One-half lived on a farm, 21% lived in the country/not on a farm and 28% lived in town. 90% of subject households had at least one bicycle. Overall, 78% had ridden a bicycle in the past year. Those from farms had lower proportions that had ridden a bicycle in the past year (73%) than those living elsewhere (83%), *p* < 0.001, and also rode them less frequently. The mean importance (rated 1–10) of wearing a bike helmet was 4.7 (median 4). Males, older teens, non-Hispanic White individuals, and those from farms all ascribed lower bicycle helmet importance than their corresponding peers. Only 15% supported laws requiring bicycle helmet use. Three-quarters (74%) rarely or never wore a helmet; only 13% said they always or mostly wore a helmet. A direct relationship was noted between helmet use and those who rode more frequently, and to those ascribing higher importance to helmet use. Only 12% stated their parents had a strict “no helmet, no riding” rule. However, those with a rule had 18 times greater odds of supporting bicycle helmet laws and had a higher median ascribed bicycle helmet importance as compared to those without a rule (9 vs. 4). Moreover, participants with a strict rule had 32 times higher odds of wearing a bicycle helmet always/most of the time versus those without a rule.

**Conclusions:**

Bicycle helmet use is infrequent among rural adolescents. Youth whose parents had a strict “no helmet, no riding” rule placed greater importance on using helmets, were more supportive of bicycle helmet laws, and had significantly greater helmet use.

## Background

Bicycling is a popular childhood leisure activity in the United States, with over half of children 3–17 years riding a bike at least once in 2018 [1]. Of those youth who had ridden a bike, the average number of bike riding days was 56 (median 28) [1]. While it serves as both recreation and a mode of transportation, bicycling also introduces significant risks. Among recreational sports, injuries due to bicycles are one of the leading causes of emergency department (ED) visits for youth [2, 3].

Suffering an injury to the brain is one of the greatest risks posed to bicyclists [4–15]. A study of National Electronic Injury Surveillance System data found head injuries accounted for 10% of ED visits and 37% of hospital admissions for injured bicyclists aged 5–14 years [12]. Nationally representative data from the Kid’s Inpatient Database showed that one-third of hospitalized bicyclists under 20 years of age suffered a head injury [13]. Moreover, traumatic brain injuries are responsible for 70–80% of pediatric bicycle-related deaths [14, 15].

Helmets are an effective intervention for preventing bicycle-related head trauma [2, 8, 10, 11, 16, 17]. Studies have found that wearing a bicycle helmet can reduce the risk of head injury by 52–85% [8, 11, 18–20]. Those dying in bicycle crashes are about 3 times less likely to be helmeted than survivors [21]. In fact, a study of nearly 3,400 bicyclists treated for head injuries in EDs, including youth, noted that the only significant factor separating survivors from those fatally injured was helmet use [17].

Despite the effectiveness of bicycle helmets in reducing severity and prevalence of head injuries, helmet use among youth remains low [18, 22–24]. Of children < 16 years admitted to the Children’s Hospital of West Virginia with bicycle injuries, 88% were not wearing a helmet [18]. In one study, less than half of parents (45%) reported that their oldest child < 16 years always wore a helmet when riding a bicycle [22]. One national study found only 25% of children and adolescents reported always wearing their helmet [25]. Helmets have been described as an inconvenience; they are a burden to locate, take too long to put on and take off, and there is often no easy place to store them [26]. However, studies have found states with laws requiring bicycle helmets for younger riders have significantly higher percentages reporting helmet use than states without such laws [2, 18, 27–32].

Research on bicycle helmet use has predominantly focused on urban settings with very little investigation of rural helmet wearing behaviors. Although rural cycling is perceived as safer than urban cycling, one study found rural youth injured on bikes had higher hospitalization rates [33]. In order to develop effective interventions to increase helmet use among adolescents in rural areas, it is important to understand the underlying attitudes and behaviors that influence helmet use. Previous studies have shown parental estimates of helmet use by their children as generally being higher than the child’s report; therefore, it is important to directly ask youth themselves in order to gather more accurate helmet usage data [27, 34]. Our study objectives were to determine rural Iowa adolescents’ frequency of bicycle helmet use, and to evaluate their attitudes regarding the importance of wearing helmets and bicycle helmet laws. In addition, we were interested in identifying demographic factors associated with the use or lack of use of helmets, and in particular, the impact of parental “no helmet, no riding” rules.

## Methods

### Study population

A cross-sectional survey involving a convenience sample of attendees at the 2022 Iowa FFA Leadership Conference in Ames, Iowa, was administered at the University of Iowa Stead Family Children’s Hospital (SFCH) injury prevention booth. We estimate that 85–90% of attendees approached near the booth chose to participate.

FFA (previously named Future Farmers of America) is an intra-curricular middle school and high school organization for students interested in agriculture and leadership. There are over 1 million FFA members throughout the fifty states, Puerto Rico and the U.S. Virgin Islands [35]. Iowa accounts for about 20,000 members in 275 chapters and are primarily from rural areas.

Participants completed a survey electronically by cell phone via a Quick Response (QR) code, or by paper which was later entered into the Qualtrics database (Qualtrics International, Incorporated, Provo, Utah). Surveys were filled out anonymously and independently, and reviewed by staff for completeness. Participants received a small prize, which was determined by a Plinko board, as an incentive to participate.

## Survey

The survey was developed by the University of Iowa SFCH Injury Prevention Program and members of their Off-Road Vehicle Task Force to help inform their injury prevention activities regarding helmets. The questions explored student’s attitudes and beliefs about helmet use on motorcycles, dirt bikes, snowmobiles, horseback riding, all-terrain vehicles (ATVs) and bicycles. The focus of this manuscript is bicycle helmet use.

Demographic variables included sex (male, female, non-binary, other), age (years), race/ethnicity (White, Black/African American, Asian, Latino/Latinx, Other), and where they lived (on a farm, in the country but not on a farm, in town). The survey queried whether the FFA member’s family owned a bicycle, their frequency of bicycle riding in the past year (daily, weekly, monthly, a few times or less, haven’t ridden in the past year), and their frequency of bicycle helmet use in the past year (always, most of the time, sometimes, rarely, never, haven’t ridden in the past year). In addition, respondents ranked the importance of helmet wearing on a scale from 1 to 10 with 1 being “not at all important” and 10 being “extremely important.” Participants also provided their opinion as to whether there should be a law that requires bicyclists to wear a helmet. Lastly, members were asked if their parents/guardians had a strict “no helmet, no riding” rule.

### Data analysis

The data were provided to the research team by the SFCH Injury Prevention Program. Since the analysis was performed on an anonymous existing database, the University of Iowa Institutional Review Board considered the study exempt. Frequencies, bivariate analyses with chi-square and Fisher’s exact test, and multivariable logistic analyses were performed using the software program, R (https://www.r-project.org/). Missing values were excluded in the analyses and were generally about 5% or less for variables.

Regarding sex, individuals answering “Non-Binary” or “Other” were excluded from analyses due to their small sample size (1%). Because of the limited diversity of the study population, race/ethnicity was dichotomized into “non-Hispanic White” and “Other”. While this resulted in significant heterogeneity in the latter, it allowed for race/ethnicity to be included in analyses. To determine difference in medians, the Wilcoxon rank sum test or Kruskal-Wallis test was used depending on the number of groups compared. Significance for statistical tests were set at a value of *p* < 0.05.

## Results

### Demographics

A total of 1,331 FFA members, 32% of the 4,222 youth attendees, ranging from 13 to 18 years of age participated in the study. Nearly three-fifths (58%) were female, and 56% were between 16 and 18 years old (Table 1). The majority of respondents (94%) were non-Hispanic White. Half lived on a farm, 21% lived in the country/not on a farm, and 28% lived in town. 90% of subject’s households had at least one bicycle. 12% reported that their parents had a strict “no helmet, no riding” rule for bicycles.

**Table 1:** Demographic variables of survey respondents at the 2022 Iowa FFA leadership conference (*N* = 1331)

Variable	*n* (Col %)^a^
Sex	
Male	543 (41)
Female	770 (58)
Nonbinary	16 (1)
Age	
13 years	66 (5)
14 years	171 (13)
15 years	337 (26)
16 years	300 (23)
17 years	272 (21)
18 years	166 (13)
Race	
Non-Hispanic White	1257 (94)
Black/African American	17 (1)
Asian	8 (1)
Latino/Latinx	35 (3)
Other	13 (1)
Residence	
Farm	670 (50)
Country/Not Farm	285 (21)
Town	376 (28)
Owned a Bicycle	
Yes	1142 (90)
No	125 (10)
Strict “No Helmet, No Riding” Rule^b^	
Yes	115 (12)
No	858 (88)

^a^The sum of n may not equal the total Group N due to missing values

^b^Of those who had ridden a bicycle in the past year (*n* = 984)

### Ridden a bicycle

Overall, 78% of respondents had ridden a bicycle in the past year (Table 2). Those who lived on farms had lower proportions that had ridden a bicycle in the past year (73%) then those that lived elsewhere (country/not on a farm, 80%; town, 85%), *p* < 0.001. Individuals whose family owned a bicycle had higher percentages that had ridden in the past year than those who did not own a bicycle (82% vs. 37%), *p* < 0.001. Logistic regression analysis showed that those living on a farm were half as likely to have ridden a bicycle in the past year as those from towns, and respondents whose family owned a bicycle had 8 times greater odds of riding a bicycle in the past year than those who did not.

**Table 2:** Bivariate and multivariable logistic regression analyses regarding whether the respondent had ridden a bicycle in the past year among survey respondents at the 2022 Iowa FFA leadership conference (*N* = 1331)

Variable	Bivariate Analysis			Logistic Regression Analysis^a^	
	Ridden Yes n (Row %)^b^	Ridden No n (Row %)^b^	p-value	aOR	95% CI
All	984 (78)	280 (22)			
Sex			0.675		
Male	403 (78)	112 (22)		1.15	0.86–1.55
Female	565 (77)	168 (23)		1.0 (ref)	
Age			0.246		
13–15 years	433 (80)	109 (20)		1.0 (ref)	
16–18 years	542 (77)	162 (23)		0.93	0.69–1.24
Race			0.270		
Non-Hispanic White	935 (78)	261 (22)		1.46	0.78–2.74
Other Races/Ethnicities	48 (72)	19 (28)		1.0 (ref)	
Residence			< 0.001		
Farm	458 (73)	172 (27)		0.51	0.35–0.73
Country/Not Farm	218 (80)	55 (20)		0.64	0.41–1.01
Town	308 (85)	53 (15)		1.0 (ref)	
Bicycle Ownership			< 0.001		
Yes	939 (82)	203 (18)		8.07	5.34–12.20
No	45 (37)	77 (63)		1.0 (ref)	

aOR: Adjusted odds ratio; CI: Confidence interval

^a^ The total number of cases used in the logistic regression model was 1,231

^b^ The sum of n for a variable may not equal the total Group N due to missing values

## Bicycle riding frequency

Of participants who had ridden in the past year, one-quarter reported riding a bicycle at least weekly, while three-quarters stated they rode about monthly or less (Table 3). Older teens had lower proportions riding at least weekly (18%) as compared to younger teen (32%), *p* < 0.001. Those from farms and from the country but not a farm also had lower proportions that rode at least weekly (21% and 23%, respectively) as compared to those from towns (31%), *p* = 0.013. Logistic regression analysis demonstrated males were 1.6 times more likely to ride a bicycle at least weekly as compared to females, and older teens were about half as likely as younger teens to ride at least weekly. Additionally, participants from farms and from the country/not a farm had odds that were about 0.6 times less likely to ride a bicycle at least weekly versus those from towns.

**Table 3:** Bivariate and multivariable logistic regression analyses regarding frequency of riding a bicycle in the past year for those who had ridden in the past year among survey respondents at the 2022 Iowa FFA leadership conference (*N* = 984)

	Bivariate Analysis			Logistic Regression Analysis^a^	
	Ridden Monthly/Few Times a Year or Less n (Row %)^b^	Ridden Daily/Weekly n (Row %)^b^	p-value	aOR	95% CI
All	742 (75)	242 (25)			
Sex			0.009		
Male	287 (71)	116 (29)		1.58	1.17–2.15
Female	445 (79)	120 (21)		1.0 (ref)	
Age			< 0.001		
13–15 years	293 (68)	140 (32)		1.0 (ref)	
16–18 years	443 (82)	99 (18)		0.45	0.33–0.61
Race			0.609		0.83–4.13
Non-Hispanic White	703 (75)	232 (25)		1.85	
Other Races/Ethnicities	38 (79)	10 (21)		1.0 (ref)	
Residence			0.013		
Farm	360 (79)	98 (21)		0.61	0.43–0.86
Country/Not Farm	168 (77)	50 (23)		0.58	0.38–0.88
Town	214 (69)	94 (31)		1.0 (ref)	
Bicycle Ownership			0.878		
Yes	709 (76)	230 (24)		0.98	0.45–2.11
No	33 (73)	12 (27)		1.0 (ref)	

aOR: Adjusted odds ratio; CI: confidence interval

^a^The total number of cases used in the logistic regression model was 960, only includes those that had ridden a bicycle in the past year

^b^The sum of n for a variable may not equal the total Group N due to missing values

## Bicycle helmet use

Three-quarters (720/979, 74%) of FFA members reported rarely or never wearing a helmet and 13% (129/979) stated they sometimes wore them. Only 13% said they always or mostly wore a helmet when riding a bicycle (Table 4). Respondents who lived on farms had lower proportions reporting wearing helmets always or most of the time (10%) as compared to those who lived in towns (14%) or in the country/not on a farm (19%), *p* = 0.004. However, multivariate analysis showed no significant difference. A higher proportion of bicycle riders whose parents had a strict “no helmet, no riding” rule reported always/most of the time wearing a helmet (66%) compared to those who didn’t have this rule (6%), *p* < 0.001. In fact, those with a strict rule were 32 times more likely to wear bicycle helmets always/most of the time versus those who had no such rule.

**Table 4:** Bivariate and multivariable logistic regression analyses regarding whether a helmet is worn for those who have ridden a bicycle in the past year among survey respondents at the 2022 Iowa FFA leadership conference (*N* = 984)

Variable	Bivariate Analysis			Logistic Regression Analysis^a^	
	Helmeted Always/Most of the Time n (Row %)^b^	Helmeted Sometimes/ Rarely/Never n (Row %)^b^	p-value	aOR	95% CI
All	130 (13)	849 (87)			
Sex			0.833		
Male	54 (13)	347 (87)		0.98	0.61–1.58
Female	72 (13)	491 (87)		1.0 (ref)	
Age			0.426		
13–15 years	62 (14)	369 (86)		1.0 (ref)	
16–18 years	67 (12)	472 (88)		0.97	0.60–1.57
Race			1.000		
Non-Hispanic White	124 (13)	807 (87)		1.57	0.46–5.33
Other Races/Ethnicitie	6 (13)	41 (87)		1.0 (ref)	
Residence			0.004		
Farm	46 (10)	411 (90)		0.78	0.44–1.39
Country/Not Farm	42 (19)	175 (81)		1.76	0.96–3.21
Town	42 (14)	263 (86)		1.0 (ref)	
Bicycle Ownership			0.129		
Yes	128 (14)	807 (86)		4.84	0.97–24.17
No	2 (5)	42 (95)		1.0 (ref)	
Riding Frequency			0.059		
Few Times a Year/Monthly	89 (12)	650 (88)		1.0 (ref)	
Weekly/Daily	41 (17)	199 (83)		1.28	0.75–2.18
Strict “No Helmet, No Riding” Rule			< 0.001		
Yes	76 (66)	39 (34)		32.17	19.37–53.43
No	52 (6)	805 (94)		1.0 (ref)	

aOR: Adjusted odds ratio; CI: Confidence interval

^a^The total number of cases used in the logistic regression model was 949, only includes those who rode a bicycle in the past year

^b^The sum of n for a variable may not equal the total Group N due to missing values

### Bicycle helmet law beliefs

Overall, 15% of respondents supported a bicycle helmet law (Table 5). Females had higher proportions supporting a helmet law as compared to males (17% vs.12%), *p* = 0.030. Younger teens as compared to older teens (17% vs.12%) had higher proportions that felt there should be a helmet law, *p* = 0.021. Individuals who had not ridden a bicycle in the past year had higher percentages supporting a helmet law versus those who had ridden (23% vs. 13%), *p* < 0.001. Individuals whose parents had a strict “no helmet, no riding” rule had higher percentages who believed there should be a bicycle helmet law as compared to those with no rule (54% vs. 7%), *p* < 0.001. After controlling for other variables, logistic regression analysis revealed that males were nearly half as likely as females to support a bicycle helmet law. Moreover, respondents whose parents had a “no helmet, no riding” rule had 18 times greater odds of supporting a bicycle helmet law than those without this rule.

**Table 5:** Bivariate and multivariable logistic regression analyses regarding whether the respondent believes there should be a helmet law in place among survey respondents at the 2022 Iowa FFA leadership conference

Variable	Bivariate Analysis			Logistic Regression Analysis^a^	
	Support Helmet Law n (Row %)^b^	Oppose Helmet Law n (Row %)^b^	p-value	aOR	95% CI
All	187 (15)	1070 (85)			
Sex			0.030		
Male	62 (12)	450 (88)		0.56	0.35–0.90
Female	122 (17)	608 (83)		1.0 (ref)	
Age			0.021		
13–15 years	93 (17)	446 (83)		1.0 (ref)	0.44–1.09
16–18 years	87 (12)	613 (88)		0.69	
Race			1.000		
Non-Hispanic White	177 (15)	1014 (85)		1.81	0.54–6.04
Other Races/Ethnicities	10 (15)	55 (85)		1.0 (ref)	
Residence			0.833		
Farm	91 (15)	536 (85)		1.04	0.61–1.78
Country/Not Farm	39 (14)	231 (86)		1.19	0.65–2.18
Town	57 (16)	303 (84)		1.0 (ref)	
Bicycle Ownership			0.862		
Yes	170 (15)	965 (85)		0.99	0.32–3.03
No	17 (14)	105 (86)		1.0 (ref)	
Ridden in Past Year^c^			< 0.001		
Never Ridden	64 (23)	215 (77)		--	--
Ridden	122 (13)	854 (88)		--	--
Riding Frequency			0.195		
Few times/Monthly	86 (12)	652 (88)		1.0 (ref)	0.69–1.93
Weekly/Daily	36 (15)	202 (85)		1.16	
Strict “No Helmet, No Riding” Rule			< 0.001		
Yes	62 (54)	52 (46)		18.11	11.20–29.30
No	59 (7)	798 (93)		1.0 (ref)	

aOR: Adjusted odds ratio; CI: Confidence interval

^a^The total number of cases used in the logistic regression model was 947, only includes those who rode a bicycle in the past year

^b^The sum of n for a variable may not equal the total Group N due to missing values

^c^Not included in logistic regression analysis

### Importance of helmet use

The mean importance (rated 1–10) of wearing a helmet on a bike was 4.7 with a median of 4 (Table 6). Males, non-Hispanic White individuals and participants living on farms rated helmet use less important than their peers, all *p* < 0.004. Members who had a strict “no helmet, no riding” rule rated bicycle helmet use more important than those without a rule (median 9 vs. 4), *p* < 0.001. The greater the helmet use, the higher the median helmet importance rating with those always wearing a helmet rating the importance at 10 and those never wearing a helmet having a median importance of 3, *p* < 0.001. Additionally, those who were in favor of a helmet law had higher median helmet importance ratings than those who were against (10 vs. 4), *p* < 0.001.

**Table 6:** Variable comparisons in the median importance ascribed to wearing a helmet while riding a bicycle among survey respondents at the 2022 Iowa FFA leadership conference

	Median Importance (1–10)^a^	*p*-value
Overall	4	--
Sex		< 0.001
Male	4	
Female	5	
Age		0.077
13–15 years	5	
16–18 years	4	
Race		0.002
Non-Hispanic White	4	
Other Races/Ethnicities	6	
Residence		0.003
Farm	4	
Country/Not Farm	5	
Town	5	
Bicycle Ownership		0.117
Yes	4	
No	4	
Ridden in Past Year		0.118
Yes	4	
No	8	
Riding Frequency^b^		0.843
Few times/Monthly	4	
Weekly/Daily	4	
Helmet Use^b^		< 0.001
Never	3	
Sometime/Rarely	5	
Most of the time	7	
Always	10	
Helmet Law		< 0.001
Support	10	
Against	4	
Strict “No Helmet, No Riding” Rule^b^		< 0.001
Yes	9	
No	4	

^a^Median importance was rated 1-10, with 1 being “not at all important” and 10 being “extremely important"

^b^Only includes those that had ridden a bicycle in the past year

## Discussion

The vast majority of rural adolescents in the study reported their household owned at least one bicycle and nearly four-fifths had ridden a bicycle in the past year. Only 13% reported wearing a bicycle helmet always or most of the time, while three-quarters rarely or never wore one. There was a direct relationship between helmet use and those who ascribed a higher importance to wearing helmets. Respondents’ median importance rating regarding helmet use was also indicative of their support for a helmet law. Although bicycle helmet use amongst rural adolescents was infrequent overall, those whose parents had a strict “no helmet, no riding” rule held greater importance to wearing helmets, expressed greater support of helmet laws and had significantly higher bicycle helmet use.

### Rural helmet use

Few studies have examined rural adolescent bicycle helmet use or their attitudes towards bicycle helmets. Although low bicycle helmet use among youth has previously been noted, our finding of 14% always/mostly wearing a helmet is lower than most other studies [1, 22–25, 27, 34]. With respect to rural versus non-rural helmet use, a Manitoba study previously found that use was lower in youth from rural areas as compared to their peers across all ages and sex [33]. In addition, we found that youth living on farms had the lowest helmet use and a lower median importance rating for helmet use than those living elsewhere.

A common misconception is that bicycling in rural areas is not as dangerous due to fewer obstacles and less traffic [36]. Rural youth’s low helmet use likely reflects this and other misconceptions, as well as other practical and/or cultural differences of rural populations. Additionally, unlike suburban and urban centers, rural areas are less likely to have special events and campaigns educating and promoting helmet use [36]. Injury prevention efforts with the goal of increasing bicycle helmet use among rural youth will need to address these issues.

### Parental influence on helmet use

Only 12% of participants stated their parents had a strict “no helmet, no riding” rule for bicycles. However, 66% of respondents who had this rule reported they always or most of the time wore a helmet as compared to just 6% of adolescents whose family did not have this rule. In fact, those with a rule were 32 times more likely to wear a helmet most or all of the time. Furthermore, youth with this home rule placed greater importance on helmet use and were more supportive of bicycle helmet laws.

Previous studies have found helmet use by youth to be strongly associated with parental rules, parental encouragement, parental involvement and parental use [15, 23, 37–40]. For example, a Swedish study found that 84% of children 8–16 years old who reported using a helmet also reported their parents told them to wear one [38]. Similar to our study, Chicago-area children with a strict rule regarding helmet use had a higher proportion wearing helmets most or all the time (88%) as compared to those with no rule (19%) [15]. Other research shows strict bicycle helmet use rules established by a parent are a strong contributing factor in a youth’s decision to wear a helmet [37, 41].

Another important factor in increasing helmet use among youth is for adults to role model this behavior themselves. Research has shown that children’s safety behaviors are more likely predicted by their parents’ safety practices as compared to having simply received safety information from their parents [42]. Therefore, it is essential that injury prevention efforts addressing bicycle helmet use in children have strong components that target parents. Incorporating a strict “no helmet, no riding” rule is an effective behavioral intervention to increase helmet use. It appears that youth whose parents prioritize safety by establishing such a rule are also more likely to support laws and public policies that enforce similar standards.

### Helmet laws

Even after controlling for other variables, rural adolescents who had a strict “no helmet, no riding” rule were more than 18 times more likely to support a bicycle helmet law than those whose families had no rule. Other demographic groups that had higher proportions supporting implementation of a helmet law included younger teens, those who had not ridden a bicycle in the past year and females. However, overall, our study revealed only 15% of rural adolescent respondents supported a helmet law.

State laws and local ordinances have been very effective in increasing helmet use including in Maryland, the first state to mandate helmet use for children [18, 27, 28, 30–32, 43]. Currently, 17 states and the District of Columbia have youth bicycle helmet laws, covering almost half of all US children < 16 years [2]. After enactment of a Georgia helmet law, reported bicycle helmet use increased from 35 to 53% [32]. Similarly, a doubling of helmet use to 49% was observed among youth in Oregon after bicycle helmet law passage [18]. A study found that children in states with laws requiring helmets had higher percentages always wearing helmets than children from states without such laws (47% vs. 39%) [27]. Most importantly, a greater decline in head injuries have been observed in areas where bicycle helmet laws were adopted [29].

### Limitations

This study was performed in Iowa, a single rural Midwestern state with a predominantly non-Hispanic White population. Thus, the generalizability of our findings to more racially and ethnically diverse populations may be limited. We also used a convenience sample of FFA members who were mainly from rural areas of Iowa and were specifically recruited at our safety booth. Thus, participants may not fully represent convention attendees overall and/or Iowa youth in general. However, participation among those approached was high, the vast majority of counties in the state were represented, and the survey was conducted primarily to evaluate rural Iowa adolescents. This study also relied on self-reported data which can be subject to bias. By allowing students to complete the surveys anonymously and independently, the social desirability effect was hopefully minimalized.

## Conclusions

Rural adolescents in our study infrequently wore bicycle helmets and those from farms had the lowest proportions wearing helmets. This may reflect the lower importance they ascribe to helmet use. Moreover, only a small percentage of participants supported bicycle helmet laws. Rural youth often have less access to helmets, fewer protective cycling regulations, and less exposure to safety education than their peers. However, we found individuals whose households had a strict “no helmet, no riding” rule placed greater importance on helmet use, were more supportive of bicycle helmet laws, and were 32 times more likely to wear a helmet than rural youth who did not have this rule. It may be wise to target parents as a major component of any injury prevention program with goals of increasing bicycle helmet use. Along with passage of bicycle helmet legislation, parents implementing and enforcing a “no helmet, no riding” rule may be the most effective pathway for decreasing pediatric bicycle-related head injuries among both urban and rural youth.

## Data Availability

Data and materials are available to other parties for research purposes after a data sharing agreement plan is agreed to and signed. Those interested should contact the corresponding author.

## Abbreviations

ATV: All-terrain vehicle
ED: Emergency department
FFA: Formerly Future Farmers of America
QR: Quick response
SFCH: Stead Family Children's Hospital
U.S.: United States
