# Correction to: Rural adolescent attitudes and use of bicycle helmets in Iowa

**DOI:** 10.1186/s40621-026-00712-2

**Published:** 2026-09-14

**Authors:** Brooke L. Askelsen, Brianna J. Iverson, Devin E. Spolsdoff, Pam J. Hoogerwerf, Brenda Vergara, Charles A. Jennissen

**Affiliations:** 1 University of Iowa College of Liberal Arts and Sciences, Iowa City, IA USA; 2 Roy J. and Lucille A. Carver College of Medicine, University of Iowa, Iowa City, IA USA; 3 Department of Emergency Medicine, Roy J. and Lucille A. Carver College of Medicine, University of Iowa, Iowa City, IA USA; 4 Injury Prevention and Community Outreach, Stead Family Children’s Hospital, University of Iowa, Iowa City, IA USA; 5 Stead Family Department of Pediatrics, Roy J. and Lucille A. Carver College of Medicine, University of Iowa, Iowa City, IA USA; 6 University of Iowa Hospitals and Clinics, 200 Hawkins Dr, Iowa City, IA 52242 USA

**Correction to: Inj. Epidemiol. 12 (Suppl 1), 41 (2025).**

**https://doi.org/10.1186/s40621-025-00596-8**

Following publication of the original article [1], errors were identified in two cited references. To ensure the clarity and accuracy of the scientific record, the authors are issuing some corrections regarding the references cited. These corrections do not affect the methods, results, or conclusions of the original article. Due to an inadvertent mistake by the authors, Reference [21] is a duplication of Reference [8] with a different title that is incorrect. Thus, Reference [21] has been removed from the original article, and all subsequent references [22–44] have been renumbered accordingly. In addition, the title of Reference [8] should have an “of” instead of an “on” with the correct title being, “A case-control study of the effectiveness of bicycle safety helmets”.

The paragraph in the Background section currently reads:

Helmets are an effective intervention for preventing bicycle-related head trauma [2, 8, 10, 11, 16, 17]. Studies have found that wearing a bicycle helmet can reduce the risk of head injury by 52–85% [8, 11, 18–21]. Those dying in bicycle crashes are about 3 times less likely to be helmeted than survivors [22]. In fact, a study of nearly 3,400 bicyclists treated for head injuries in EDs, including youth, noted that the only significant factor separating survivors from those fatally injured was helmet use [17].

The paragraph in the Background section should read:

Helmets are an effective intervention for preventing bicycle-related head trauma [2, 8, 10, 11, 16, 17]. Studies have found that wearing a bicycle helmet can reduce the risk of head injury by 52–85% [8, 11, 18–20]. Those dying in bicycle crashes are about 3 times less likely to be helmeted than survivors [21]. In fact, a study of nearly 3,400 bicyclists treated for head injuries in EDs, including youth, noted that the only significant factor separating survivors from those fatally injured was helmet use [17].

The References currently read:

^8^ Thompson RS, Rivara FP, Thompson DC. A case-control study on the effectiveness of bicycle safety helmets. N Engl J Med. 1989;320(21):1361–7.

^21^ Thompson RS, Rivara FP, Thompson DC. Prevention of head injury by bicycle helmets: a field study of efficacy. N Engl J Med. 1989;320:1361–7.

The Reference should read:

^8^ Thompson RS, Rivara FP, Thompson DC. A case-control study of the effectiveness of bicycle safety helmets. N Engl J Med. 1989;320(21):1361–7.

The paragraph within the Background section where reference [21] was cited and the reference list have been updated in this correction article and the original article [1] has been corrected.
